# Incidence of Cellulitis Following Acupuncture Treatments in Taiwan

**DOI:** 10.3390/ijerph16203831

**Published:** 2019-10-11

**Authors:** Shun-Ku Lin, Jui-Ming Liu, Pin-Hsuan Wang, Sheng-Ping Hung, Ren-Jun Hsu, Heng-Chang Chuang, Po-Hung Lin

**Affiliations:** 1 Department of Chinese Medicine, Taipei City Hospital, Renai Branch, Taipei 106, Taiwan; gigilaskl@gmail.com (S.-K.L.); u102022004@cmu.edu.tw (P.-H.W.); haopinhe707@gmail.com (S.-P.H.); 2 Institute of Public Health, National Yang-Ming University, Taipei 112, Taiwan; 3 Division of Urology, Department of Surgery, Taoyuan General Hospital, Ministry of Health and Welfare, Taoyuan 330, Taiwan; mento1218@gmail.com (J.-M.L.); chuang20110617@yahoo.com.tw (H.-C.C.); 4 Department of Medicine, National Yang-Ming University, Taipei 112, Taiwan; 5 Graduate Institute of Life Sciences, National Defense Medical Center, Taipei 114, Taiwan; hsurnai@gmail.com; 6 Department of Chinese Medicine, China Medical University, Taichung 404, Taiwan; 7 Cancer Medicine Center of Buddhist Hualien Tzu Chi Hospital, Tzu Chi University, Hualien 970, Taiwan; 8 Department of Pathology and Graduate Institute of Pathology and Parasitology, Tri-Service General Hospital, National Defense Medical Center, Taipei 114, Taiwan; 9 Division of Urology, Department of Surgery, Chang Gung Memorial Hospital at Linkou, Taoyuan 333, Taiwan; 10 Graduate Institute of Clinical Medical Science, College of Medicine, Chang Gung University, Taoyuan 333, Taiwan

**Keywords:** acupuncture, cellulitis, risk factors, varicose veins, liver cirrhosis, heart failure

## Abstract

*Background*: Cellulitis is a complication of acupuncture, but the risk factors and annualized incidence remain unclear. Objective: This study analyzed the incidence and risk factors of cellulitis related to acupuncture in a cohort of one million participants derived from Taiwan’s Longitudinal Health Insurance Database. *Methods*: We tracked this cohort between 1997 and 2012 and recorded all outpatient medical information including diagnosis and treatment. Patients were categorized according to age, gender, comorbidities, residential area, and number of acupuncture treatments. We compared the incidence and risk of cellulitis between different demographics and comorbidities by logistic regression analysis and adjusted odds ratio (aOR) with a 95% confidence interval (95% CI). *Results*: We included 407,802 patients and 6,207,378 acupuncture treatments. The incidence of cellulitis after acupuncture was 64.4 per 100,000 courses of acupuncture treatment. The most common sites of cellulitis after acupuncture were the legs, feet, and face. Comorbidity was associated with post-acupuncture cellulitis; a multivariate logistic regression analysis showed that chronic kidney disease (aOR, 1.71; 95% CI, 1.55–1.88), rheumatoid arthritis (aOR, 1.86; 95% CI, 1.21–3.60), liver cirrhosis (aOR, 1.23; 95% CI, 1.15–1.32), diabetes mellitus (aOR, 1.69; 95% CI, 1.57–1.82), stroke (aOR, 1.44; 95% CI, 1.31–1.58), varicose veins (aOR, 2.38; 95% CI, 2.17–2.84), or heart failure (aOR, 1.81; 95% CI, 1.65–1.98) significantly increased cellulitis. Repeated exposure to acupuncture treatment was associated with an increased risk of cellulitis. *Conclusions*: A variety of chronic diseases may increase the risk of cellulitis after acupuncture. Physicians asked about past medical history before acupuncture might help to reduce cellulitis.

## 1. Introduction

Cellulitis after acupuncture is a complication that can worsen into necrotizing fasciitis, osteomyelitis, and septic shock. Patients with cellulitis often receive antibiotic treatment, with an increase in the patient’s hospitalization rate and prolonged treatment periods of the disease [1]. Common pathogenic bacteria include Streptococcus aureus, Klebsiella pneumonia, and Streptococcus agalactiae [2]. Many research teams have reported the side effects and adverse effects of acupuncture in several countries [1,2,3] and found that experienced acupuncturists and careful disinfection before application could reduce the incidence of adverse events after acupuncture [4,5]. However, these studies are based on the voluntary reports of acupuncturists. Research based on national data established by the government is still lacking, and the incidence and risk factors of cellulitis after acupuncture remain unclear. A variety of chronic diseases, including chronic kidney disease, liver cirrhosis, and diabetes mellitus, can weaken the immune system and increase the risk of infection [6]. Many patients undergoing acupuncture treatment have the disorders mentioned above, but the association between the patient’s comorbidity disease and cellulitis is still unknown. Our research aim is to investigate the incidence of cellulitis after acupuncture, the distribution of the sites of the disease, and possible risk factors.

## 2. Methods

### 2.1. Data Source

This is a retrospective cohort study with a nested case-control study. The research passed review by the Research Ethics Committee at Taipei City Hospital (review number TCHIRB-10701103-E). We used the Longitudinal Health Insurance Database 2005 (LIHD 2005) as our data source, as this database has been widely used as research material for traditional Chinese medicine [7,8]. Regions used in the study include Taiwan’s main island and offshore island regions (Kinmen, Matsu, Penghu) for the period from 1997 to 2012.

### 2.2. Study Population

**Figure 1:**
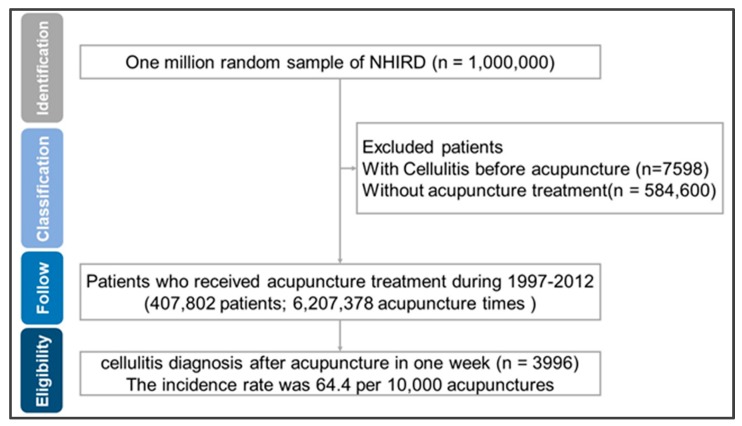
Flowchart of the recruitment of patients with cellulitis after acupuncture. We enrolled 407,802 patients with 6,207,378 acupuncture treatments and recorded 3996 cases of cellulitis that resulted in hospitalization and treatment with antibiotics form the National Health Insurance Research Database (NHIRD). Abbreviations: NHIRD, National Health Insurance Research Database.

The research flowchart is shown in Figure 1. We included 407,802 patients with 6,207,378 acupuncture treatments between 1997 and 2002. The insurance records of acupuncture treatments (procedure codes: B41–B46) are registered in the “Details of ambulatory care orders file” of LHID 2005, including all outpatient medical procedures. Due to the comprehensiveness of data in LHID 2005, we can review all medical data for one million patients from 1997 to 2012 and confirm the number of times, frequency, and treatment areas of patients who underwent acupuncture treatment.

### 2.3. Cellulitis after Acupuncture

We collected data from patients that were hospitalized due to cellulitis within 2 weeks of acupuncture treatment. Any hospitalization records more than 2 weeks from a patient’s time of acupuncture were discarded because those cellulitis cases may have been caused by other factors. We excluded patients who sought medical attention for cellulitis (*n* = 7598) in the month prior to acupuncture treatment. Additionally, we excluded patients who underwent invasive medical procedures in the month prior to being diagnosed with cellulitis; this included surgery, injection, debridement, suture, and tooth extraction. We used diagnostic standards from the International Classification of Diseases, Ninth Revision, Clinical Modification (ICD-9-CM), including diagnoses such as ICD-9-CM 681 (Cellulitis and abscess of finger and toe) and ICD-9-CM 682 (Other cellulitis and abscess). The hospitalization records of all patients were recorded in the “Inpatient expenditures by admissions” file, and we only selected hospitalization records where cellulitis was listed among the top 3 of diagnoses. In order to ensure diagnostic accuracy, we targeted the position of cellulitis according to specific codes (681.0 finger; 681.1 toe; 682.0 face; 682.1 neck; 682.2 trunk; 682.3 upper arm and forearm; 682.4 hand, except fingers and thumb; 682.5 buttocks; 682.6 leg, except foot; 682.7 foot, except toes) for ICD-9-CM. We compared acupuncture location with the location of cellulitis diagnosis and excluded noncompliant data to maintain the correctness of the diagnosis.

### 2.4. Possible Confounder

Cellulitis is a clinically common infectious disease, with many interference factors that can impact its occurrence rate. We considered a patient’s individual factors, including age, gender, location, and income. Patients were categorized into four groups depending on the age they were when diagnosed with cellulitis (<20 years, 20–40 years, 40–60 years, and >60 years). The patient’s gender was categorized according to data logged into health insurance, and any patients lacking age or gender data were excluded from the study. Patients were categorized by region using the insurance location from NHI, as this location gave a rough representation of the patient’s place of residence and work. This type of categorization method has already been broadly applied in Chinese medicine research [9], and we separated insured regions into four levels according to the degree of urbanization: very high, high, moderate, and low. The degree of urbanization was stratified according to five factors: the population density (people/km^2^), the proportion of people with college educational levels or above, the proportion of people over 65 years, the proportion of agricultural workers, and the number of physicians per 100,000 people. The degree of urbanization can encompass a region’s medical resources, average age, and conditions of environmental exposure; it is a good predictive factor for inclination to accept Chinese medicine treatments [10]. We used insurance payments to categorize patients into four tiers: no salary and dependent on others guaranteed, $1–19,999 New Taiwan dollars (NT, one US dollar = NT $30), $20,000–39,999 NT, and >$40,000 NT. This categorization method is broadly applied in health insurance research in Taiwan [11,12].

The patient’s disease comorbidity might also affect the incidence of cellulitis. Diseases that damage the skin barrier, impede the flow of lymph and veins, and reduce sensory acuity may increase the risk of cellulitis. We included the following diseases into the confounders following previous researches findings: chronic kidney disease (ICD-9-CM: 585,586), liver cirrhosis (ICD-9-CM: 571), diabetes mellitus (ICD-9-CM: 250), dementia (ICD-9-CM: 290, 294, and 331.0), Parkinson’s disease (ICD-9-CM: 332), stroke (ICD-9-CM: 430, 431, 432, and 434), varicose veins (ICD-9-CM: 454), heart failure (ICD-9-CM: 428), and autoimmune diseases (Supplementary Table S1) [13,14].

To understand the relationship between acupuncture frequency and cellulitis, we calculated the number of acupuncture sessions administered to patients and classified them as <6, 6–30, and >30 courses. If patients receive less than six courses, we classified those patients as not regularly treated with acupuncture. We classified patients with >30 acupuncture treatments courses as high-use patients.

### 2.5. Statistical Analysis

We calculated the incidence rate and annualized incidence rate of cellulitis between 1997–2012. The differences between post-acupuncture cellulitis patients and other acupuncture patients, including areas like demographics and medical features, were identified using a chi-square test. In order to reduce the impact of the confounders in this study, the propensity score was used to match the two groups of patients according to age (5 years for each tier), gender, place of insurance, insurance payment tier, number of acupuncture treatments (permissible difference of positive/negative 2), and the hospital or clinic where acupuncture treatment was received. The odds ratio (OR) of cellulitis and 95% confidence intervals (95% CI) were calculated using multivariate logistic regression, with a *p*-value <0.05 to mark statistical significance and a *p*-value <0.001 marked as high significance. For matched groups, we used conditional logistic regression to calculate the odds ratio. Our calculation of a crude odds ratio and the adjusted odds ratio (aOR) were based on 2 different models: Model 1 corrected demographic variables, including age, gender, urbanization, and insured amount; model 2 corrected demographic variables and disease comorbidities involving chronic kidney disease, rheumatoid arthritis, liver cirrhosis, diabetes mellitus, dementia, Parkinson’s disease, stroke, varicose veins, and heart failure. We utilized SAS statistical software (version 9.4; SAS Institute Inc., Cary, NC, USA) to process data and perform a statistical analysis.

## 3. Results

We included 407,802 patients and 6,207,378 acupuncture treatments in this study. Patients were hospitalized and treated with antibiotics in 3996 cases of cellulitis. The incidence of cellulitis after acupuncture was 64.4 times per 100,000 courses of acupuncture treatment.

**Figure 2:**
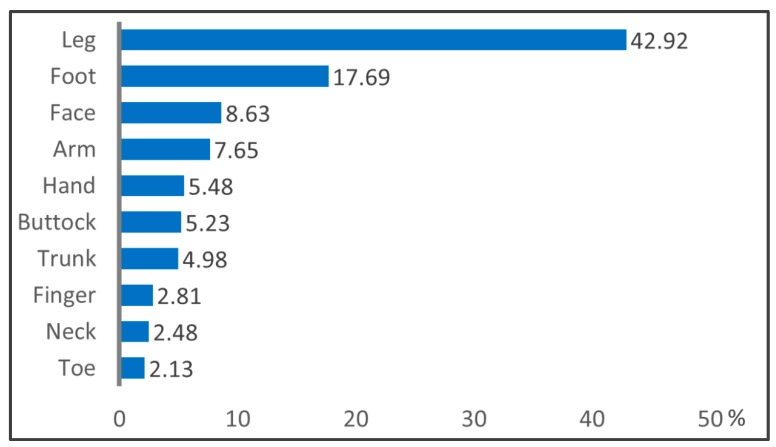
The proportion of cellulitis in different parts of the body. The leg and foot are the most prone to cellulitis after acupuncture, accounting for 42.95% and 17.69%, respectively; on the other hand, the trunk, fingers, and neck feature the lowest proportion.

Figure 2 demonstrates the proportion of cellulitis in different parts of the body. The disease is mostly found in the leg (42.95%) and foot (17.69%). On the other hand, the trunk, fingers, and neck feature the lowest proportion, the total of the three only accounts for about 10%.

Table 1 shows the demographic and medical feature of patients with cellulitis after acupuncture. Patients with cellulitis had a higher proportion of more than 60 years, a male gender, a low amount of insurance, and lived in a low urbanization area compared to their counterparts without cellulitis. Cellulitis sufferers also showed a higher prevalence of comorbid diseases and more courses of acupuncture treatment. After a propensity score matching process was done four times, there was no significant difference of age, gender, urbanization, the insured amount, and courses of acupuncture treatment between patients with or without cellulitis, but the comorbidity proportion was still higher in the groups with cellulitis.

We present the association between demographic/medical factors and the risk of cellulitis after acupuncture in Table 2. The age of a patient is related to their risk of cellulitis, with an adjusted odds ratio of 1.98 (1.60–2.43) and 2.89 (2.34–3.58) for ages 40–60 and over 60. The higher insured amount and urbanization of insured area were related to lower cellulitis risk. The patient’s disease comorbidity was associated with post-acupuncture cellulitis. A multivariate logistic regression analysis showed that chronic kidney disease (aOR, 1.66; 95% CI, 1.51–1.84), autoimmune diseases (aOR, 1.39; 95% CI, 1.27–1.53), liver cirrhosis (aOR, 2.05; 95% CI, 1.80–2.34), diabetes mellitus (aOR, 1.71; 95% CI, 1.59–1.84), dementia (aOR, 1.35; 95% CI, 1.20–1.52), stroke (aOR, 1.44; 95% CI, 1.31–1.58), varicose veins (aOR, 2.48; 95% CI, 2.16–2.85), or heart failure (aOR, 1.76; 95% CI, 1.60–1.93) might significantly increase the risk of cellulitis after adjusting for demographic variables.

The more acupuncture treatment the patients receive, the higher the risk of cellulitis. Compared to the patients who received less than six courses of acupuncture, the adjusted odds were 1.79 (1.66–1.92) and 2.68 (2.46–2.93) for patients with six to thirty courses and more than 30 courses.

We analyzed the effects of multiple comorbidities on the risk of cellulitis after acupuncture in the propensity matching groups. The results are shown in Table 3. The estimated value of the Adjusted odds ratio decreased significantly in each variable compared to the pre-corrected results. Chronic diseases including chronic kidney disease, autoimmune diseases, liver cirrhosis, diabetes mellitus, dementia, Parkinson’s disease, stroke, varicose veins, and heart failure are still significantly associated with post-acupuncture cellulitis after correction.

## 4. Discussion

This study used the National Health Insurance Research Database (NHIRD) to explore the risk and possible risk factors of cellulitis in acupuncture treatments. When patients suffer from chronic diseases, such as chronic kidney disease, rheumatoid arthritis, liver cirrhosis, diabetes mellitus, stroke, dementia, varicose veins, or heart failure, there is an increased risk of cellulitis after acupuncture treatments. Additionally, other risk factors may include undergoing more acupuncture treatments, being male, old age, and inhabiting areas with a lower degree of urbanization. The proportion of cellulitis after acupuncture increased with age and patients over 60 years old account for 42.5%. We recommend traditional doctor to be more careful with older patients.

Our study discovered that the incidence rate of cellulitis after acupuncture treatment was approximately 64.4 per 100,000 acupuncture treatments. The incidence rate of cellulitis after acupuncture treatment in this study is lower than that in past studies. One possible factor may be that we only included severe cases of cellulitis, such as patients who required hospitalization and antibiotic treatment. A more stringent evaluation standard can reduce the interference of information bias. In this study, we used data from the NHIRD to accurately evaluate each acupuncture treatment of patients, the number of treatments, and whether the patient has previously been diagnosed with chronic disease. Regardless of whether cellulitis patients returned for treatment at the hospital where they received acupuncture or did not notify the doctor who performed their acupuncture treatment, researchers can find their medical records in NHIRD. This database covers more than 99% of Taiwan’s population and medical institutions, ensuring that misclassification bias and selection bias are minimized.

The incidence rate of cellulitis after acupuncture treatments seems to show an increasing trend with time, possibly due to a greater acceptance of acupuncture treatments among Taiwan’s general public, thereby causing more severe or multiple chronic disease patients to accept acupuncture treatments. These results indicate the importance of infection control measures. All doctors who practice traditional Chinese medicine in Taiwan must pass a national exam and regularly undergo infection control training. However, continued training (including washing hands correctly and disinfection) and building a medical environment that emphasizes infection control remains a topic that requires immediate attention [15].

Our study demonstrated that multiple chronic diseases could lead to a higher risk of cellulitis after an acupuncture treatment, including chronic kidney disease, rheumatoid arthritis, liver cirrhosis, diabetes mellitus, stroke, dementia, varicose veins, or heart failure. Liver cirrhosis decreases a patient’s immune function and poses a higher risk of infection from drug-resistant pathogens [16]; additionally, liver cirrhosis patients with cellulitis have a higher risk of complications, such as acute renal failure [17]. Chronic kidney disease and diabetes mellitus are risk factors of cellulitis that can lead to more severe symptoms, such as necrotizing fasciitis. Rheumatoid arthritis patients undergoing long-term corticosteroid and non-steroidal anti-inflammatory drug treatments with lowered resistance against infection have a higher risk of infection through injury [18]. Patients with varicose veins suffering from symptoms such as bloating, redness of the skin, pain, and itchy calves may give patients difficulty in identifying early signs of infection, leading to delayed treatment and cellulitis that is more severe.

Additionally, poor circulation in the lower limbs and lymphatic reflux will increase the risk of infection [19]. Stroke and dementia patients suffer from damaged sensory function in their bodies, sometimes making it hard to detect the early signs of infection. These symptoms can lead to a higher risk of cellulitis [20].

We suggest that doctors practicing traditional Chinese medicine carefully interview patients on their medical history, especially the aforementioned chronic diseases, and determine if they have sought treatment for severe cellulitis in the past before administering acupuncture treatment. For high-risk patients, we suggest a consultation with doctors specializing in infectious disease before acupuncture and close monitoring of any infection symptoms, including fever, local pain, warmth, swelling, redness, or hyperemia.

This study suffered certain limitations. First, the patient examination results, including complete blood count, white blood cell differential count, C-reactive protein, and bacteriological culture data, do not appear in the NHIRD database. Therefore, we have no way of knowing the types of bacterial infection and their severity. Past studies include a detailed discussion of pathogenic bacteria in cellulitis after acupuncture treatment [2]. Second, this study is a retrospective generation study, and the details of acupuncture treatment, such as depth, name of the acupuncture point, treatment time, and combined use of moxibustion, are not included in the NHIRD database. We expect random double-blind studies to offer better explanations of the causal relationships between chronic diseases and infection from acupuncture treatment. Third, this study only includes the general population who are residents from Taiwan; it is possible that the results of this study may not be extrapolated to other regions of the world.

## 5. Conclusions

Our results suggest that the incidence of post-acupuncture cellulitis is about 64.4 per 100,000 acupuncture treatments, and the most prone areas of cellulitis occurrence are the leg and foot. A variety of chronic diseases may increase the risk of cellulitis after acupuncture. A physician’s assessment of past medical history before acupuncture might help to reduce risk of cellulitis.

## Figures and Tables

**Table 1:** Demographic and medical feature of patients with cellulitis after acupuncture.

Variables	Cellulitis Patients No. (%)	All Patients without Cellulitis No. (%)	*p*-Value of Chi-Square Test	1:4 Matching Patients without Cellulitis No. (%)	*p*-Value of Chi-Square Test
**Total**	3996 (100.0%)	407,802 (100.0%)	-	15,984 (100.0%)	
**Age at diagnosis**			<0.0001		0.67
<20	116 (2.9%)	40,768 (10.0%)	-	451 (2.8%)	
20–40	859 (21.5%)	157,152 (38.5%)	-	3381 (21.2%)	
40–60	1322 (33.1%)	143,472 (35.2%)	-	5271 (33.0%)	
≥60	1699 (42.5%)	66,410 (16.3%)	-	6881 (43.0%)	
**Gender**			<0.0001		1.00
Female	1839 (46.0%)	217,814 (53.4%)	-	7356 (46.0%)	
Male	2157 (54.0%)	189,988 (46.6%)	-	8628 (54.0%)	
**Urbanization**			<0.0001		0.75
Very high	1796 (44.9%)	204,226 (50.1%)	-	7152 (44.7%)	
High	1015 (25.4%)	103,250 (25.3%)	-	3948 (24.7%)	
Moderate	806 (20.2%)	73,017 (17.9%)	-	3316 (20.7%)	
Low	379 (9.5%)	27,309 (6.7%)	-	1568 (9.8%)	
**Insured amount (NT$)**			<0.0001		0.79
Dependent	1035 (25.9%)	155,239 (38.1%)	-	4039 (25.3%)	
$1–19,999	2153 (53.9%)	161,563 (39.6%)	-	8382 (52.4%)	
$20,000–39,999	573 (14.3%)	61,244 (15.0%)	-	2231 (14.0%)	
≥$40,000	235 (5.9%)	29,756 (7.3%)	-	1332 (8.3%)	
**Comorbidity**			-		
Chronic kidney disease	607 (15.2%)	15,947 (3.9%)	<0.0001	1343 (8.4%)	<0.05
Autoimmune diseases	666 (16.7%)	28,473 (7.0%)	<0.0001	1522 (9.5%)	<0.05
Liver Cirrhosis	277 (6.9%)	7298 (1.8%)	<0.0001	687 (4.3%)	<0.05
Diabetes mellitus	1755 (43.9%)	77,716 (19.1%)	<0.0001	3596 (22.5%)	<0.05
Dementia	447 (11.2%)	12,407 (3.0%)	<0.0001	975 (6.1%)	<0.05
Parkinson’s disease	218 (5.5%)	6551 (1.6%)	<0.0001	1646 (10.3%)	<0.05
Stroke	750 (18.8%)	23,311 (5.7%)	<0.0001	751 (4.7%)	<0.05
Varicose veins	247 (6.2%)	6333 (1.6%)	<0.0001	943 (5.9%)	<0.05
Heart failure	751 (18.8%)	19,320 (4.7%)	<0.0001	2270 (14.2%)	<0.05
**Acupuncture treatment courses**			<0.0001		0.81
<6	1567 (39.2%)	247,625 (60.7%)	-	6251 (39.1%)	
6–30	1546 (38.7%)	121,621 (29.8%)	-	6172 (38.6%)	
≥30	883 (22.1%)	38,556 (9.5%)	-	3561 (22.3%)	

**Table 2:** The association between demographic/medical factors and cellulitis after acupuncture in all acupuncture groups.

Variables	Odds Ratio (95% Confidence Interval)		
	Crude Odds Ratio	Adjusted Model 1	Adjusted Model 2
**Age at diagnosis**			
<20	[Reference]	[Reference]	[Reference]
20–40	1.92 (1.58–2.33) *	1.62 (1.33–1.98) *	1.62 (1.32–1.97) *
40–60	3.24 (2.68–3.92) *	1.98 (1.61–2.44) *	1.98 (1.60–2.43) *
≥60	8.99 (7.45–10.86) *	2.90 (2.34–3.58) *	2.89 (2.34–3.58) *
**Gender**			
Female	[Reference]	[Reference]	[Reference]
Male	1.35 (1.26–1.43) *	1.57 (1.47–1.68) *	1.57 (1.47–1.68) *
**Urbanization**			
Very high	[Reference]	[Reference]	[Reference]
High	1.13 (1.04–1.22) *	1.09 (1.01–1.18) *	1.08 (1.06–1.17) *
Moderate	1.26 (1.16–1.37) *	1.19 (1.09–1.30) *	1.19 (1.09–1.29) *
Low	1.59 (1.42–1.78) *	1.35 (1.20–1.51) *	1.35 (1.20–1.51) *
**Insured amount (NT$)**			
Dependent	[Reference]	[Reference]	[Reference]
$1–19,999	2.00 (1.86–2.15) *	1.18 (1.09–1.29) *	1.18 (1.08–1.28) *
$20,000–39,999	1.40 (1.27–1.56) *	0.92 (0.82–1.03)	0.91 (0.81–1.02)
$≥40,000	1.19 (1.03–1.37) *	0.73 (0.63–0.85) *	0.73 (0.62–0.85) *
**Comorbidity**			
Chronic kidney disease	1.87 (1.70–2.06) *	1.67 (1.51–1.84) *	1.66 (1.51–1.84) *
Autoimmune diseases	1.36 (1.10–1.68) *	1.41 (1.28–1.54) *	1.39 (1.27–1.53) *
Liver Cirrhosis	2.31 (2.03–2.63) *	2.05 (1.80–2.34) *	2.05 (1.80–2.34) *
Diabetes mellitus	1.98 (1.85–2.12) *	1.72 (1.59–1.85) *	1.71 (1.59–1.84) *
Dementia	1.50 (1.34–1.69) *	1.35 (1.21–1.52) *	1.35 (1.20–1.52) *
Parkinson’s disease	1.11 (0.95–1.29) *	1.00 (0.86–1.17)	0.99 (0.85–1.16)
Stroke	1.69 (1.54–1.85) *	1.45 (1.32–1.59) *	1.44 (1.31–1.58) *
Varicose veins	2.58 (2.25–2.96) *	2.48 (2.16–2.85) *	2.48 (2.16–2.85) *
Heart failure	2.03 (1.86–2.23) *	1.77 (1.61–1.94) *	1.76 (1.60–1.93) *
**Acupuncture treatment courses**			
<6	[Reference]	[Reference]	[Reference]
6–30	1.84 (1.72–1.98) *	1.79 (1.67–1.93) *	1.79 (1.66–1.92) *
≥30	2.80 (2.58–3.05) *	2.69 (2.47–2.93) *	2.68 (2.46–2.93) *

* *p* < 0.05.

**Table 3:** The association between medical factors and cellulitis after acupuncture in propensity matching groups.

Comorbidity	Odds Ratio (95% Confidence Interval)	
	Crude Odds Ratio	Adjusted Odds Ratio
Chronic kidney disease	1.84 (1.67–2.03) *	1.62 (1.47–1.79) *
Autoimmune diseases	1.44 (1.22–1.70) *	1.38 (1.17–1.62) *
Liver Cirrhosis	2.26 (1.98–2.57) *	2.00 (1.75–2.28) *
Diabetes mellitus	2.06 (1.92–2.21) *	1.71 (1.59–1.84) *
Dementia	1.49 (1.33–1.67) *	1.32 (1.18–1.48) *
Parkinson’s disease	1.14 (0.98–1.33)	1.02 (0.87–1.19)
Stroke	1.75 (1.60–1.92) *	1.47 (1.34–1.62) *
Varicose veins	2.74 (2.39–3.13) *	2.55 (2.22–2.92) *
Heart failure	2.02 (1.84–2.22) *	1.71 (1.56–1.88) *

* *p* < 0.05.

## Supplementary Materials

The following are available online at https://www.mdpi.com/1660-4601/16/20/3831/s1, Table S1: ICD-9 diagnostic codes of 33 autoimmune diseases.
