# Supplementary material for: Incidence of Cellulitis Following Acupuncture Treatments in Taiwan
Source: Int J Environ Res Public Health. 2019 Oct 11;16(20):3831. doi: 10.3390/ijerph16203831 (PMC6843534; doi:10.3390/ijerph16203831)
Supplement: Supplementary file 1 [file ijerph-16-03831-s001.pdf]

Table S1. ICD-9 diagnostic codes of 33 autoimmune diseases

Autoimmune Diseases	ICD-9-CM Codes
Graves' disease	242,242.01
Crohn's disease	555.0, 555.1, 555.2, 555.9
Psoriasis	696, 696.1, 696.8
Systemic lupus erythematosus	710.0
Rheumatoid arthritis	714
Ankylosing spondylitis	720.0
Guillain–Barre´syndrome	357.0
Sjogren's syndrome	710.2
Myasthenia gravis	358.0
Pernicious anemia	281.0
Hereditary hemolytic anemia	282
Polyarteritis nodosa	446
Celiac disease	579.0
Uveitis	364.00, 364.01
Polymyalgia rheumatica	725
Dermatomyositis	710.3
Hashimoto's thyroiditis	245.2
Hypersensitivity vasculitis	446.2, 446.29
Behcet's disease	136.1
Polymyositis	710.4
Alopecia areata	704.01
Wegener's granulomatosis	446.4
Ulcerative colitis	556.0, 556.6, 556.8, 556.9
Autoimmune hemolytic anemia	283.0
Pemphigus	694.4
Multiple sclerosis	340
Systemic sclerosis	710.1
Juvenile rheumatoid arthritis	714.30, 714.33
Goodpasture syndrome	446.21
Giant cell arteritis	446.5
Thromboangitis obliterans	443.1
Arteritis obliterans	446.7
Kawasaki disease	446.1

Abbreviations: ICD-9, International Classification of Diseases-9th revision
